# Objective understanding of Nutri-Score Front-Of-Package nutrition label according to individual characteristics of subjects: Comparisons with other format labels

**DOI:** 10.1371/journal.pone.0202095

**Published:** 2018-08-23

**Authors:** Manon Egnell, Pauline Ducrot, Mathilde Touvier, Benjamin Allès, Serge Hercberg, Emmanuelle Kesse-Guyot, Chantal Julia

**Affiliations:** 1 Sorbonne Paris Cité Epidemiology and Statistics Research Center (CRESS), U1153 Inserm, U1125, Inra, Cnam, Paris 13 University, Nutritional Epidemiology Research Team (EREN), Bobigny, France; 2 Santé publique France, the national public health agency, Saint-Maurice, France; 3 Public health department, Avicenne Hospital, Bobigny, France; College of Agricultural Sciences, UNITED STATES

## Abstract

**Background:**

To improve nutritional status and prevent chronic nutrition-related diseases, international organizations have recommended the use of multiple strategies, including front-of-package nutrition labelling (FOPL). In France, the Nutri-Score has been selected by health authorities in March 2017. However, to be effective in purchasing situations, the label needs to be well understood, which may be influenced by label format and sociodemographic characteristics. This study aimed at investigating the objective understanding of the Nutri-Score compared to other label formats, and more particularly among specific at-risk populations.

**Methods:**

The objective understanding of four FOPLs—namely Nutri-Score, Multiple Traffic Lights (MTL), the simplified nutrition labelling system (SENS), and modified Reference Intakes (mRIs)—was investigated in a sample from the NutriNet-Santé French cohort (n = 3,751). Logistic regression mixed models were computed to assess the association of the four FOPLs, compared to a “no label” situation, on the consumers’ ability to rank products according to their overall nutritional quality. Objective understanding was also investigated according to sociodemographic and lifestyle characteristics.

**Results:**

Compared to a “no label” situation, all FOPLs were significantly associated to an increase in consumers’ ability to classify the products correctly, with wide disparities in results according to formats. The best performance was observed for the Nutri-Score (OR = 20.33(17.68–23.37)), followed by SENS (OR = 9.57(8.50–10.77)), MTL (OR = 3.55(3.20–3.93)) and mRIs (OR = 1.53(1.39–1.69)). This ranking was similar among all sub-populations and the ORs associated to the Nutri-Score were over 10, whichever the sub-group considered. Women, younger people, non-smokers, individuals with higher educational level and those with children had a higher capacity to identify healthier products (all P≤0.05).

**Conclusion:**

Nutri-Score, with a summarized graded and color-coded format, using semantic colours, is associated to a higher objective understanding than monochrome and nutrient-specific labels. Furthermore, though objective understanding may differ according to individual characteristics of subjects, the magnitude of effect of the Nutri-Score largely outweighed this effect, even in the at-risk populations.

## Introduction

Considering the burden attributable to nutrition-related diseases [1], public health policies aim to improve the nutritional status of populations and more widely to prevent the onset of chronic diseases [2]. One potential strategy to improve diet quality of consumers is to encourage healthier food choices at the point of purchase and allow them to integrate nutrition in their food choices [3]. However, it has been demonstrated that current nutritional information on food packs is difficult to read and understand for consumers in many European countries, and most of them do not use this information during their purchases [4]. Indeed, while 40% of consumers report looking at nutritional information during purchases [5], only less than 10% actually do so in observational in-store studies [6]. Therefore front-of-pack (FOP) nutrition labels have been developed and applied on pre-packaged food products, to increase consumer awareness of the nutritional quality of food and improve consumer information. Indeed, this measure has been proven to help consumers identifying healthier food products [7–17]. Additionally, FOP nutrition labels are suggested to encourage the food industry to develop products of higher nutritional quality through innovation or reformulation [18,19].

In 2017, French health authorities selected the Nutri-Score as a voluntary front-of-pack labelling for pre-packaged foods. The Nutri-Score is a summary graded label, providing a single assessment of the nutritional quality of the food. The Nutri-Score was selected after a series of experimental and ‘real-life’ studies pertaining on perception [20], understanding [21] and food purchases [22–27], which showed that this format was comparatively more efficient than other formats currently available in the world, in particular in vulnerable populations.

According to the theoretical framework proposed by Grunert et al., a FOP label should retain attention and then be accepted and understood by the consumer to potentially influence his food choices [9]. It has been suggested that in addition to the label format the objective understanding of a label is influenced by sociodemographic characteristics. Some studies have shown that color-coded labels were more adapted to improve consumer awareness, especially among individuals with low socioeconomic status, educational level, knowledge in nutrition and adherence to dietary recommendations [4,6–10,14,21,28]. However, few studies have assessed comparatively the objective understanding of summary graded formats, while they may have positive effects on consumer behaviour [10,29]. Hence it appeared of importance to investigate the objective understanding of the Nutri-Score compared to other FOP label formats, especially in sub-groups with specific sociodemographic characteristics.

This study aimed at investigating the objective understanding of Nutri-Score compared to different FOP labels’ formats, using the consumer’s ability to rank a sample of French products according to nutritional quality. In addition, the performance of each FOP label format among specific sub-populations was assessed, in particular according to socio-demographic and lifestyle characteristics.

## Materials and methods

### Study population

The NutriNet-Santé study is a French ongoing web-based cohort launched in May 2009 [30]. Briefly, the objective of the NutriNet-Santé study is to investigate the associations between nutrition and health, as well as the determinants of dietary behaviours and nutritional status. Volunteers participants aged over 18 years with access to the Internet are recruited by vast multimedia campaigns, and have to fill a set of online questionnaires, assessing dietary intakes, physical activity, anthropometric characteristics, lifestyle, socioeconomic conditions and health status. The NutriNet-Santé study is conducted according to the French Institute for Health and Medical Research (IRB Inserm n°0000388FWA00005831) and the "Commission Nationale de l’Informatique et des Libertés" (CNIL n°908450/n°909216). Electronic informed consent was obtained from each participant. The questionnaire pertaining to the objective understanding of the labels was sent to a randomly selected fraction of the study population (N = 7000). Response to the questionnaire was optional for participants, and the questionnaire was available for completion for 3 months. A version of the questionnaire is available on the following link: https://info.etude-nutrinet-sante.fr/.

### Design

#### FOP label formats tested

**Fig 1:**
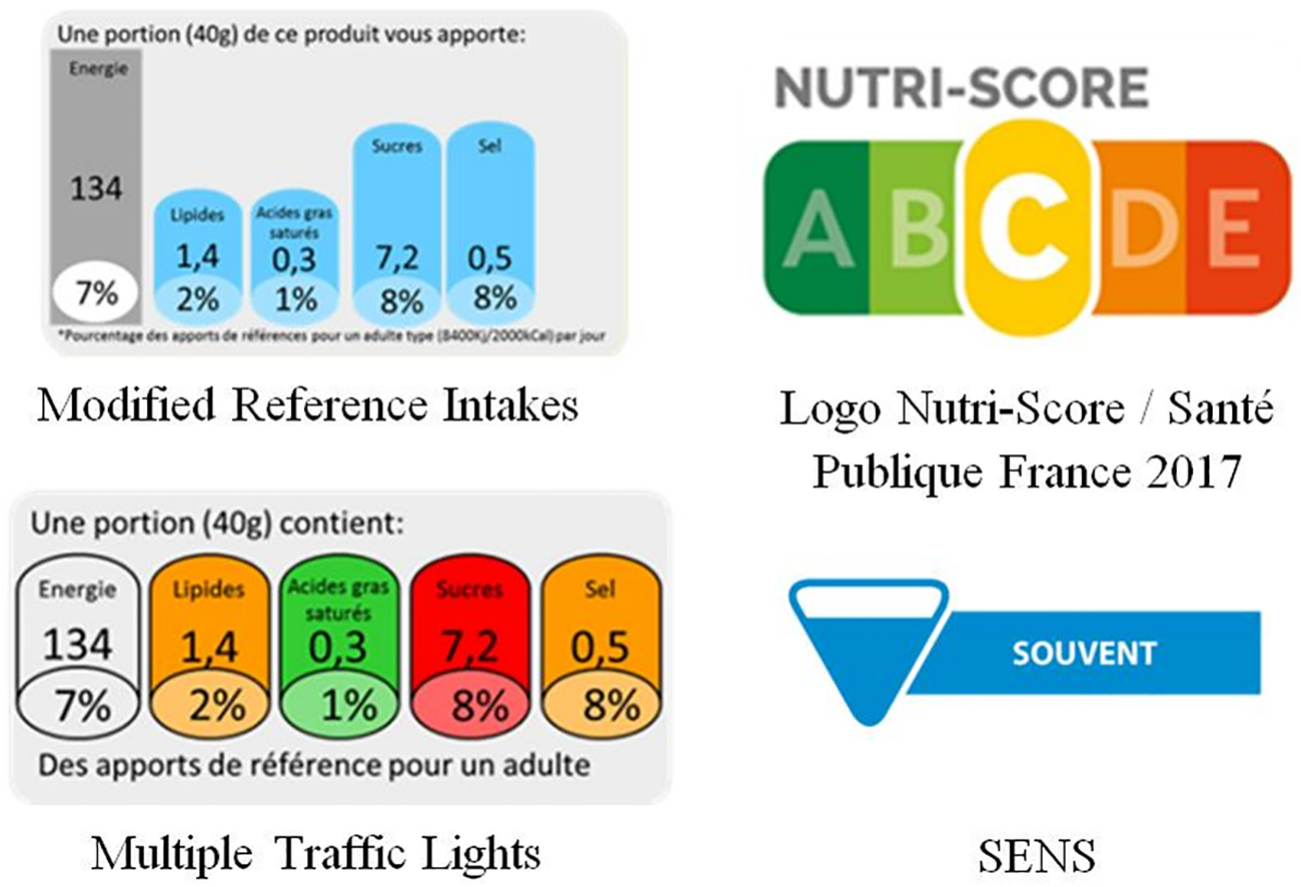
Front-Of-Package labels tested.

Four label formats were tested in the present study (Fig 1): Nutri-Score (official FOP label from the French Ministry of Health); SENS, a graded summary FOP label indicating information on consumption frequency and proposed in 2014 by the Trade and Retail Federation [31]; a modified version of the Reference Intakes (mRIs) proposed by industry and the MTL.

The two summary FOP label formats included in the present study were:

(1) The Nutri-Score, based on the Food Standard Agency (FSA) nutrient profiling system, indicates the overall nutritional quality of a given food item [32]. This label is represented by a scale of five colours, from green indicating food products with the highest nutritional quality to red for products with lower nutritional quality, with corresponding letter (from A on green to E on red). For each food item, colour is based on its FSA score, modified by High Council for Public Health (HCSP) [33]: green (-15 to -1 points), light green (0 to 2 points), yellow (3 to 10 points), orange (11 to 18 points) and red (19 points and over). This label has been chosen by health authorities to be applied on food products in the French market in March 2017 [34].
(2) The SENS label is a four-category graded summary label and indicates the recommended consumption frequency of a given food item, based on a modified version of the SAIN, LIM algorithm [35]. Each frequency is associated with a colour: green-labelled food could be eaten “very often”, blue “often”, orange “regularly in small quantities” and purple “occasionally or in small quantities” [31].

The nutrient-specific formats investigated in the study were the following two:

(3) The modified Reference Intakes (mRIs) label, is a modified version of the Guideline Daily Amounts, and indicates the kilocalories and the amount of fat, saturated fatty acids, sugars and sodium in gram per portion, and their contribution (in percentage for each nutrient) to the reference intakes [36]. The mRIs represents the percentage of reference intakes for a portion in the form of histograms.
(4) Multiple Traffic Lights (MTL) label, introduced by the UK FSA, provides information on the nutrient amounts for a portion of product, regarding total fat, saturated fatty acids, sugars and sodium. Depending on the amount of the different nutrient in the product (low, moderate, high), a colour is attributed to each nutrient (green, orange, red respectively). Colour codes were assigned according to FSA criteria [37].

A situation with no FOP label on the food package was used as a reference.

#### Procedure

Objective understanding of FOP labels’ formats was assessed in October 2016 using an online questionnaire, under five different conditions: four corresponding to the four tested FOP labels’ formats and one alternative with no label.

Participants were shown pictures of three products from the same food category, and were asked to rank the products from the lowest to the highest nutritional quality according to the information provided (FOP label or control condition with no label). For the ranking task, subjects could choose among the following answers: “lowest nutritional quality”, “intermediate nutritional quality”, “highest nutritional quality” and “I don’t know”.

Food categories tested in the study were chosen following two main objectives: (i) the category had to be commonly consumed in the French population, and (ii) it had to include a large diversity of products in terms of nutritional quality within the categories. Thus, five different food categories, representing various eating occasion (breakfast, snacks, meals) were tested: breakfast cereals, pre-prepared dishes, sandwiches, canned fish and sweet biscuits. In each food category, the three products proposed had differing nutritional quality, thus enabling the ranking task using the FOP label. Products were selected so that the correct ranking was the same for all FOP labels. No other information on nutritional facts was given and all other labels were removed from the pictures (e.g. “organic”, “fair trade”, etc).

To avoid potential effects of the food category upon understanding of the FOP label (i.e. knowledge on a specific product), each label was applied on each product category. All participants were shown five combinations of label and product, where all four FOP label conditions (and control situation) and five food categories were represented. To control for potential order effect of the labels, a rotation system based on a Latin square design was used to ensure that an equal number of participants were shown each combination. For example, a subject was shown the Nutri-Score on breakfast cereals and SENS on sandwiches, while another was shown the Nutri-Score on sweet biscuits and SENS on breakfast cereals, etc. Furthermore, rotation was also applied to the order of the presented labels (e.g. the Nutri-Score was the first FOP label presented for some participants, while others were shown SENS first).

Objective understanding was assessed using the results of the ranking task. The ranking task was considered as correct when participants ranked the three products in the right order, according to the information on the nutritional quality provided by the FOP label. The ranking was considered as incorrect when the participant made at least one mistake or answered “I don’t know”.

### Data collection

#### Sociodemographic and lifestyle data

At inclusion and annually, thereafter, participants were invited to complete a questionnaire related to sociodemographic and lifestyle data [30,38], including sex, age, educational level, income per consumption unit [39] (one consumption unit (CU) is attributed for the first adult of the household, 0.5 CU for other persons aged 14 or older and 0.3 CU for children under 14 years old), household composition, smoking status, as well as a physical activity questionnaire (computed following International Physical Activity Questionnaire recommendations [40]) and a questionnaire on anthropometrics measurements [41,42] (e.g. height, weight).

#### Dietary data and adherence to dietary recommendations

Dietary intakes were assessed at baseline and every six months using a series of three non-consecutive validated web-based self-administered 24h-dietary records, randomly distributed over a 2-week period (2 weekdays and 1 weekend day) [41,43,44]. Participants used a dedicated interface to declare all foods and beverages they consumed during the day, and the portion sizes, using validated photographs [45]. Nutrient intake was estimated using a published French food composition table [46]. Dietary underreporting was identified on the basis of the method proposed by Black, using the basal metabolic rate and Goldberg cut-off, and under-energy reporters were excluded [47].

A modified version of the PNNS guidelines score was used to assess adherence to dietary recommendations, the PNNS-GS (“Programme National Nutrition Santé”- Guideline Score) based on the French national nutritional guidelines and previously described in details [48]. Briefly, this 15-point score includes eight components on food serving recommendations and four on moderation in consumption [49]. The thirteenth component on adherence to physical activity was not included in the modified version of the PNNS-GS (mPNNS-GS). In addition, overconsumption was taking into account with a penalty when individuals had higher energy intakes compared to estimated expenditure [48]. A higher mPNNS-GS reflects better adherence to French nutritional recommendations. The mPNNS-GS was used as a proxy of the nutritional quality of the diet.

### Statistical analyses

Analysis was performed on data of participants who had filled the questionnaire on FOP labels. Participants who answered “I don’t know” to more than two-thirds of the items were excluded from analyses. Chi-square tests were performed to compare included and excluded subjects.

For all covariates, there were no missing data except for physical activity (0.7%), educational level (1.0%), income (11.2%) and mPNNS-GS (13.8%), for which multiple imputation was performed [50]. Percentages of correct answers were calculated for each FOP label, across sub-groups of population and chi-square tests were performed, except for categories of body mass index where Fisher test were performed. The association between the ability to rank correctly the three products (“yes” or “no”, dependent variable) and FOP label formats and individuals characteristics (independent variables) was evaluated using logistic regression mixed models with a random intercept. Individual characteristics were sex, age (18–29, 30–49, 50–64, ≥65 years), educational level (up to secondary, some college or university degree), income per consumption unit (<1200€/month, 1200–1800€, 1800–2700€ and >2700€/month), smoking status (current smokers/non- or former smokers), physical activity (high, moderate, low), household composition (with or without children), body mass index, and mPNNS-GS (in quartiles). Variables displaying a significance level of p<0.15 in univariate models were included in the multivariate model.

Interactions between individual characteristics of participants and FOP label format were assessed. To take account of multiple comparisons, a p-value of 10^−3^ was considered statistically significant, except in interaction analyses, where a p-value <0.15 was considered significant, given the low power of interaction tests. Analyses were carried out with SAS software (version 9.4; SAS Institute, Inc.).

## Results

A total of 4,328 participants completed the FOP label questionnaire. 577 subjects, who did not answer or responded “I don’t know” to at least two-thirds of the items were excluded from the analyses, leading to a sample of 3,751 participants (mean age: 49.4±14.4 years, 81.4% of women). Characteristics of included and excluded subjects are presented in S1 Table. Included participants were more likely to be women, middle-aged, physically active, non-overweight, never smokers, without children, and to have higher educational level and income.

Table 1 displays the percentages of correct answers for each label format, across sub-groups of populations. The presence of any of the FOP labels was characterized by an increase of the number of correct answers compared to the “no label” situation. In the Nutri-Score situation, almost 90% of participants reached the correct answer. Irrespective of the sub-group of population, the rate of correct answers was significantly higher for the Nutri-Score, with a rate over 83%, followed by SENS, MTL, and mRIs. The differences observed between the “no label” situation and a FOPL situation (in particular for Nutri-Score) were much higher than the differences observed across population subgroups in any given FOPL situation.

**Table 1:** Percentage of correct answers of each FOP label, across sub-groups of population (N = 3,751).

	N (%)	Nutri-Score	SENS	MTL	mRIs	No label	P*
**Total of correct answer**		90.32	82.03	65.48	47.80	38.87	<0.0001
**Category of product**							
Breakfast cereals	3740 (99.71)	93.04^a^	87.43^b^	74.26^c^	56.14^d^	57.40^d^	<0.0001
Pre-prepared dishes	3736 (99.60)	91.40^a^	85.27^b^	82.32^b^	77.72^c^	33.71^d^	<0.0001
Sandwiches	3738 (99.65)	88.86^a^	75.98^b^	62.38^c^	43.37^d^	34.31^e^	<0.0001
Canned fish	3738 (99.65)	87.71^a^	80.39^b^	64.85^c^	31.59^d^	32.46^d^	<0.0001
Sweet biscuits	3739 (99.68)	90.61^a^	81.56^b^	42.60^c^	30.21^e^	35.07^d^	<0.0001
**Sex**							
Men	698 (18.61)	88.70^a^	78.11^b^	61.80^c^	46.49^d^	33.33^e^	<0.0001
Women	3053 (81.39)	90.70^a^	82.93^b^	66.33^c^	48.12^d^	40.12^e^	<0.0001
**Age (years)**							
18–29	265 (7.06)	94.34^a^	87.92^b^	77.36^c^	56.60^d^	45.66^e^	<0.0001
30–49	1595 (42.52)	93.17^a^	87.08^b^	71.47^c^	50.16^d^	40.63^e^	<0.0001
50–64	1128 (30.07)	90.17^a^	79.63^b^	61.03^c^	47.12^d^	37.38^e^	<0.0001
≥65	763 (20.35)	83.22^a^	73.00^b^	55.44^c^	40.89^d^	34.99^e^	<0.0001
**Educational level**							
Up to secondary	505 (13.46)	84.16^a^	73.47^b^	56.44^c^	40.99^d^	35.84^d^	<0.0001
Some college	477 (12.72)	88.89^a^	79.66^b^	60.17^c^	44.44^d^	39.83^d^	<0.0001
University degree	2730 (72.78)	91.94^a^	84.21^b^	68.28^c^	49.63^d^	39.45^e^	<0.0001
Missing data	39 (1.04)	75.00^a^	70.00^ab^	52.50^b^	50.00^b^	25.00^c^	<0.0001
**Monthly income per consumption unit (€/CU)**							
<1200	240 (6.40)	89.04^a^	79.76^b^	63.41^c^	48.90^d^	43.17^d^	<0.0001
1200–1800	554 (14.77)	89.59^a^	82.27^b^	67.51^c^	46.91^d^	39.47^e^	<0.0001
1800–2700	497 (13.25)	90.68^a^	82.69^b^	64.15^c^	48.39^d^	38.73^e^	<0.0001
>2700	2041 (54.41)	91.29^a^	82.78^b^	67.01^c^	46.47^d^	37.03^e^	<0.0001
Missing data	419 (11.17)	90.69^a^	81.62^b^	63.48^c^	49.88^d^	36.04^e^	<0.0001
**Household composition**							
Adults only	2521 (67.21)	89.61^a^	81.36^b^	64.30^c^	47.04^d^	38.87^e^	<0.0001
Adults and children	1230 (32.79)	91.79^a^	83.41^b^	67.89^c^	49.35^d^	38.86^e^	<0.0001
**Smoking status**							
Current smoker	561 (14.96)	89.84^a^	85.03^b^	68.09^c^	47.06^d^	40.82^e^	<0.0001
Former smoker	1246 (33.22)	87.64^a^	77.93^b^	61.08^c^	47.03^d^	38.92^e^	<0.0001
Never smoker	1944 (51.82)	92.18^a^	83.80^b^	67.54^c^	48.51^d^	38.27^e^	<0.0001
**Physical activity level**							
High	1137 (30.31)	89.53^a^	80.83^b^	60.42^c^	45.29^d^	39.05^e^	<0.0001
Moderate	1658 (44.20)	90.35^a^	82.93^b^	68.58^c^	49.76^d^	38.60^e^	<0.0001
Low	929 (24.77)	91.28^a^	82.02^b^	66.20^c^	47.15^d^	38.86^e^	<0.0001
Missing data	27 (0.72)	89.29^a^	78.57^ab^	64.29^bc^	57.14^c^	46.43^c^	0.006
**Body Mass Index (kg/m**^**2**^**)**							
<18.5	209 (5.57)	91.39^a^	85.17^b^	66.51^c^	47.85^d^	44.02^d^	<0.0001
18.5–24.9	2370 (63.18)	91.14^a^	83.08^b^	67.59^c^	47.05^d^	39.20^e^	<0.0001
25–29.9	810 (21.59)	89.38^a^	78.40^b^	61.17^c^	49.63^d^	36.67^e^	<0.0001
30–34.9	266 (7.09)	87.22^a^	80.83^b^	59.40^c^	49.25^d^	37.22^e^	<0.0001
35–39.9	68 (1.82)	83.82^a^	83.82^a^	58.82^b^	42.65^b^	42.65^b^	<0.0001
≥40	28 (0.75)	85.71^a^	82.14^ab^	60.71^bc^	57.14^c^	42.86^c^	0.003
**mPNNS-GS quartiles**							
Quartile 1	811 (21.62)	90.84^a^	82.92^b^	66.58^c^	42.70^d^	37.87^e^	<0.0001
Quartile 2	812 (21.65)	90.76^a^	83.87^b^	69.21^c^	48.15^d^	37.56^e^	<0.0001
Quartile 3	819 (21.83)	89.15^a^	80.85^b^	63.90^c^	46.10^d^	43.17^d^	<0.0001
Quartile 4	792 (21.12)	90.05^a^	80.98^b^	62.22^c^	52.14^d^	36.90^e^	<0.0001
Missing data	517 (13.78)	91.32^a^	81.95^b^	65.65^c^	48.65^d^	38.45^e^	<0.0001

MTL, Mutliple Traffic Lights; mRIs, modified Reference Intakes; mPNNS-GS, modified “Programme National Nutrition Santé”–Guideline Score; CU, Consumption Unit. One CU is attributed for the first adult of the household, 0.5 CU for other persons aged 14 or older and 0.3 CU for children under 14 years old. Percentages of correct answers with the same letter were not significantly different.

* P-values are based on chi square or Fisher tests.

The association between product ranking ability and individual characteristics of participants and label formats is shown in Table 2. Regarding sociodemographic characteristics, and consistent with results of the cross-tabulations (Table 1), the odds of correct product ranking in multivariate model were greater among women, young participants and those with higher educational level. However, the odds of correctly ranking products did not vary according to household income or nutritional quality of the diet (mPNNS-GS). In both univariate and multivariate models, the odds of correct product ranking were increased in FOP label conditions compared to “no label” situation. Among the four formats, the Nutri-Score performed best (OR = 20.33 (17.68–23.37)), followed by SENS (OR = 9.57 (8.50–10.77)), MTL (OR = 3.55 (3.20–3.93)) and mRIs (OR = 1.53 (1.39–1.69)). Furthermore, the odds ratios corresponding to the FOP label effects were much higher than the odds corresponding to the sociodemographic characteristics effects.

**Table 2:** Association between FOP label formats and objective understanding using logistic regression mixed models in univariate and multivariate analyses (N = 3,751)^a^.

	Univariate	*P*	Multivariate^b^	*P*
	OR (95% CI)		OR (95% CI)	
**FOP labels**				
**No label**	1		1	
**Nutri-Score**	17.94 (15.67–20.53)	<0.0001	20.33 (17.68–23.37)	<0.0001
**SENS**	8.51 (7.59–9.54)	<0.0001	9.57 (8.50–10.77)	<0.0001
**MTL**	3.32 (3.00–3.67)	<0.0001	3.55 (3.20–3.93)	<0.0001
**mRIs**	1.49 (1.36–1.64)	<0.0001	1.53 (1.39–1.69)	<0.0001
**Sex**				
**Men**	1		1	
**Women**	1.19 (1.10–1.29)	<0.0001	1.15 (1.03–1.27)	0.01
**Age (years)**				
**18–29**	1		1	
**30–49**	0.83 (0.73–0.95)	0.005	0.81 (0.69–0.97)	0.02
**50–64**	0.65 (0.57–0.74)	<0.0001	0.63 (0.53–0.75)	<0.0001
**≥65**	0.51 (0.45–0.59)	<0.0001	0.47 (0.39–0.57)	<0.0001
**Educational level**				
**Up to secondary**	1		1	
**Some college**	1.19 (1.06–1.34)	0.004	1.11 (0.95–1.29)	0.2
**University degree**	1.45 (1.32–1.58)	<0.0001	1.33 (1.18–1.51)	<0.0001
**Monthly income per consumption unit (€/CU)**				
**>1800**	1			
**1200–1800**	1.00 (0.90–1.12)	0.9		
**1800–2700**	0.98 (0.88–1.10)	0.7		
**>2700**	0.98 (0.89–1.08)	0.7		
**Household composition**				
**Adults only**	1		1	
**Adults and children**	1.09 (1.02–1.17)	0.008	0.97 (0.88–1.06)	0.5
**Smoking status**				
**Current smoker**	1		1	
**Former smoker**	0.85 (0.77–0.94)	0.001	0.96 (0.85–1.10)	0.6
**Never smoker**	1.00 (0.91–1.09)	0.9	1.02 (0.90–1.15)	0.8
**Physical activity level**				
**High**	1		1	
**Moderate**	1.14 (1.06–1.23)	0.0004	1.04 (0.94–1.14)	0.5
**Low**	1.10 (1.01–1.19)	0.03	1.00 (0.89–1.11)	0.9
**Category of product**				
**Breakfast cereals**	1		1	
**Pre-prepared dishes**	1.06 (0.95–1.18)	0.3	1.03 (0.92–1.16)	0.6
**Sandwiches**	0.55 (0.50–0.61)	<0.0001	0.45 (0.40–0.51)	<0.0001
**Canned fish**	0.52 (0.47–0.58)	<0.0001	0.42 (0.38–0.47)	<0.0001
**Sweet biscuits**	0.45 (0.40–0.49)	<0.0001	0.35 (0.31–0.39)	<0.0001
**Body Mass Index (kg/m**^**2**^**)**				
**<18.5**	1		1	
**18.5–24.9**	0.94 (0.82–1.08)	0.4	1.05 (0.88–1.26)	0.6
**25–29.9**	0.84 (0.73–0.98)	0.03	1.03 (0.84–1.25)	0.8
**30–34.9**	0.83 (0.69–0.99)	0.04	1.01 (0.80–1.28)	0.9
**35–39.9**	0.81 (0.62–1.06)	0.1	0.95 (0.67–1.34)	0.8
**≥40**	0.94 (0.64–1.39)	0.8	1.07 (0.65–1.76)	0.8
**mPNNS-GS**				
**Quartile 1**	1			
**Quartile 2**	1.06 (0.97–1.15)	0.2		
**Quartile 3**	1.00 (0.92–1.09)	1		
**Quartile 4**	0.99 (0.82–1.07)	0.8		

OR, Odds Ratio; CI, Confidence Interval; MTL, Mutliple Traffic Lights; mRIs, modified Reference Intakes; mPNNS-GS, modified “Programme National Nutrition Santé”–Guideline Score; CU, Consumption Unit. One CU is attributed for the first adult of the household, 0.5 CU for other persons aged 14 or older and 0.3 CU for children under 14 years old.

^a^ The modelled probability was correct ranking of the three products according to their nutritional quality.

^b^ Multivariable model was adjusted on sex, age, educational level, household composition, smoking status, physical activity level, category of product and body mass index (variables which displayed a significance level of p<0.15 in univariate models).

Statistically significant interactions between FOP label formats and age (p<0.0001), educational level (p<0.0001), household composition (p = 0.15) and smoking status (p = 0.002) were observed. Stratified analyses showed that the effect of the FOP labels varied across socio-demographic sub-groups. However, if the magnitude of effect varied in the different sub-groups for each FOP label, the relative ranking across labels was similar in all sub-groups (Table 3). Compared to the “no label” situation, all FOP labels increased the ability of participants to rank the products according to their nutritional quality. However, the Nutri-Score was the format which showed better results, whatever the sub-group (lowest OR = 12.40 (9.48–16.21) among older people and highest OR = 26.99 (21.92–33.22) among never smokers), followed by SENS (lowest OR = 6.40 (4.71–8.69) among participants with low educational level and highest OR = 12.69 (10.44–15.42) among middle-aged people), MTL (lowest OR = 2.58 (2.06–3.23) among older people and highest OR = 4.58 (3.07–6.83) among younger participants) and mRIs (lowest OR = 1.28 (0.97–1.68) among participants with intermediate and low educational level and highest OR = 1.66 (1.40–1.97) among participants living in a household with children).

**Table 3:** Multivariable^a^ logistic regression mixed model of the association between objective understanding and FOP label formats, across sub-groups at risk (N = 3,751).

	No label	Nutri-Score	SENS	MTL	mRIs	P-value	P interaction
		OR (95% CI)	OR (95% CI)	OR (95% CI)	OR (95% CI)		
**Age (years)**							
**18–29**	1	23.19 (12.75–42.18)	10.14 (6.34–16.23)	4.58 (3.07–6.83)	1.58 (1.11–2.25)	<0.0001	<0.0001
**30–49**	1	25.61 (20.25–32.39)	12.69 (10.44–15.42)	4.41 (3.75–5.19)	1.57 (1.35–1.82)	<0.0001	
**50–64**	1	22.22 (17.21–28.69)	8.77 (7.10–10.84)	3.04 (2.53–3.67)	1.60 (1.34–1.92)	<0.0001	
**≥65**	1	12.40 (9.48–16.21)	6.45 (5.06–8.22)	2.58 (2.06–3.23)	1.32 (1.06–1.65)	<0.0001	
**Educational level**							
**Up to secondary**	1	12.98 (9.25–18.23)	6.40 (4.71–8.69)	2.62 (1.98–3.46)	1.28 (0.97–1.69)	<0.0001	<0.0001
**Some college**	1	15.51 (10.76–22.35)	7.67 (5.60–10.50)	2.59 (1.96–3.42)	1.28 (0.97–1.68)	<0.0001	
**University degree**	1	23.79 (20.04–28.23)	10.84 (9.40–12.50)	3.96 (3.50–4.48)	1.63 (1.45–1.83)	<0.0001	
**Household composition**							
**Adults only**	1	19.17 (16.21–22.66)	9.26 (8.02–10.69)	3.35 (2.95–3.80)	1.47 (1.30–1.66)	<0.0001	0.15
**Adults and children**	1	23.06(17.91–29.67)	10.21 (8.29–12.58)	3.97 (3.31–4.76)	1.66 (1.40–1.97)	<0.0001	
**Smoking status**							
**Current smoker**	1	18.28 (12.76–26.19)	11.28 (8.16–15.58)	3.71 (2.82–4.88)	1.38 (1.07–1.78)	<0.0001	0.002
**Former smoker**	1	14.66 (11.73–18.31)	6.97 (5.74–8.46)	2.80 (2.35–3.34)	1.46 (1.23–1.73)	<0.0001	
**Never smoker**	1	26.99 (21.92–33.22)	11.47 (9.68–13.59)	4.00 (3.54–4.75)	1.63 (1.42–1.87)	<0.0001	

OR, Odds Ratio; CI, Confidence Interval; MTL, Mutliple Traffic Lights; mRIs, modified Reference Intakes; mPNNS-GS, modified “Programme National Nutrition Santé”–Guideline Score.

^a^ Multivariate models were adjusted on sex, age, household composition, smoking status, physical activity and food category.

## Discussion

In this study, we observed that all FOP labels increased the ability of consumers to rank the products according to their nutritional quality, compared to a control situation without any FOP label. Furthermore, nutrition labels had a stronger influence on the ability of identifying healthier food products than individual characteristics of participants. However, results differed between the label formats. Indeed, the Nutri-Score showed much better results, with odds ratios over 20 (and more than 80% of correct answers, whichever the population group), followed by SENS, MTL and mRIs, for which odds ratios were lower than 10. Similar trends were observed in sub-groups using stratified analyses on sociodemographic characteristics. The objective understanding of each FOP label format was however higher among young, non-smokers, participants with higher educational level and for households with children.

Our results are consistent with a similar study conducted in the NutriNet-Santé cohort [21], which used a different set of label formats, and in particular, a previous graphical version of the Nutri-Score, the 5-Colour Nutrition Label (5-CNL). In both studies, the 5-CNL/Nutri-Score outperformed all other graphical formats, including MTL and Guidelines Dietary Amounts. Our study expands these results, showing that the Nutri-Score also outperforms other summary graded formats such as the SENS. Moreover, odds ratios associated with the Nutri-Score appear higher than those observed with the 5-CNL, showing that the updated graphical format may have a higher objective understanding.

The mRIs was the FOP label with the lower objective understanding, which could be explained by various elements. First, it may be due to its monochrome format with nutrient amounts expressed only as percentage and grams, and secondly to the evaluation system per portion. Studies have demonstrated that nutrient-specific labels that only emphasize numeric information can be confusing to consumers, especially for individuals with low educational level or social classes [4,6,10,14,21,51]. In comparison, nutrient-specific formats incorporating text and symbolic colours, such as MTL, seem to be better understood and increase consumer ability to identify healthier food products [10,14,16,20,21,52–55]. In the present study, we observed that MTL was better understood than mRIs label, but the magnitude of effect was lower compared to summary formats, as it was outperformed by both Nutri-Score and SENS. Indeed, we noted that nutrition labels with summary formats, such as the Nutri-Score and SENS, were much better understood by consumers, in all sub-groups of population, with the highest performance for the Nutri-Score. The elevated objective understanding of the Nutri-Score could be explained by two main elements. First, this format provides graded summarized information about the overall nutritional quality of the food, through a single indicator limiting potential confusion on nutrition terms [56]. Secondly, it combines colours and text, which has been demonstrated to be easier to read and understand [10,14,20,21]. Thus, despite the use also of semantic colours in the MTL label, the gradual and summarized format of the Nutri-Score was far better understood. The SENS label, which displays similar characteristics to the Nutri-Score, was less effective to help consumers discriminating product healthiness. This result may be partly explained by the use of non-semantic colours (i.e. blue and purple), while semantic colours as green and red may influence consumer’s perception and behaviour. Indeed, it has been demonstrated that green may be associated to healthfulness and healthy eating [57] and red might influence conscious awareness [58]. Moreover, the SENS label incorporates a consumption frequency, which may require further processing by the consumer. Indeed, though there is a relationship between consumption frequency, portion size and the nutritional quality of food products, the two dimensions may not be directly linked in consumers, which may have impacted results. Furthermore, in a study assessing perception [59], the SENS format was less appreciated than the Nutri-Score, and more likely to be perceived as guilt-laden. Conversely, the Nutri-Score was considered as quick to process and easy to understand by a majority of participants [59]. As a favourable perception is one of the pre-requisites for label use, our results suggest that it may also be associated with a higher objective understanding.

In our study, similar trends were observed for the understanding of FOP label formats across population sub-groups. However, some disparities in the performance of each format were detected across sub-groups of populations. For each nutrition label, the ability to rank products according to the information provided was lower among older participants and those with lower educational level. These results are in line with other studies, which have observed that older people and individuals with lower educational level had more difficulties to understand FOP labels [6,7,16,51,53,60]. We observed that the objective understanding of FOP labels was higher among never smokers. This may be explained by the greater interest in nutritional information and labels of these individuals compared to smokers or former smokers [61]. This finding is in line with other studies, which have noted that former or never smokers are more likely to read nutrition labels [61–65]. We also found that households with children were more likely to correctly rank the food products. Our findings may be due to the fact that the presence of children in the household might increase concerns of parents and motivate nutrition information search behaviours. Indeed, reviews suggested that larger household and those with children were more likely to search for nutrition information and use nutritional labels in general [4,66], even if studies observed that household size may be inversely associated to label use [67,68].

In the present study, we found that individual characteristics of participants, including notably age and educational level, were associated to the ability of products’ ranking. Women were also more able to rank the product according to the nutritional quality, that may be explained by their greater interest in nutrition [9] and the fact they are more likely to use nutrition labels [4,7,9,66]. However, the associated odds ratios for sociodemographic variables were much lower than those for FOP labels, showing that FOPLs outweigh individual characteristics of subjects in objective understanding.

The strengths of this study pertained to the heterogeneity of the population in terms of socio-demographic characteristics, enabling us to carry out analyses across sub-groups. In addition, to prevent the potential bias of label’s order, a rotation system was used, and to control for potential bias of food product, all combination of food category and FOP label were tested. Furthermore, a set of three products was used to fit more realistic conditions and to limit the risk of ‘positive’ rankings due to random answers.

Some limitations should be acknowledged. First, caution is needed regarding the extrapolation of these results to the entire French population. Indeed, this study involved participants from NutriNet-Santé, a volunteer cohort with overall more health-conscious behaviour and higher socio-professional and educational level. Moreover, the questionnaire was optional for participants and available during a short period of time (3 months), yielding a response rate of 62%, which may have increased selection bias. Compared with excluded participants, included subjects were more often women and had a higher educational level. Moreover, results may be influenced by the subjective choice of the food products and categories. Indeed, the familiarity with some food categories may have led to a higher number of correct answers in the given food category. However, the design of the questionnaire ascertained that an equal number of participants were shown each food category/label combination, and the analyses were carried out comparatively with a “no label” situation, therefore limiting the extent of this bias. Finally, participants did not have access to the nutrition facts normally available on the back of package during the ranking procedure, making the task harder especially in “no label” situation.

In conclusion, the results of the present study show that FOP nutrition labels had a stronger influence on the ability of consumers to identify healthier products than individual characteristics of participants. The Nutri-Score, with a summarized graded and colour-coded format using semantic colours, was associated to a higher objective understanding than other formats, including the nutrient-specific Multiple Traffic Lights label. Moreover, the effect of the Nutri-Score clearly outweighed the differences observed across socio-demographic sub-groups, showing its applicability to vulnerable populations. Given these results, the Nutri-Score should be considered as a valid alternative for FOP nutrition labelling.

## Supporting information

S1 Table Individual characteristics of included (N = 3,751) and excluded (N = 577) participants. (PDF)

## Abbreviations

FOP: Front-Of-Package
FSA: Food Standard Agency
MTL: Multiple Traffic Lights
mRIs: modified Reference Intakes
mPNNS-GS: modified “Programme National Nutrition Santé”—Guideline Score
