# Supplementary material for: Objective understanding of Nutri-Score Front-Of-Package nutrition label according to individual characteristics of subjects: Comparisons with other format labels
Source: PLoS One. 2018 Aug 23;13(8):e0202095. doi: 10.1371/journal.pone.0202095 (PMC6107140; doi:10.1371/journal.pone.0202095)
Supplement: S1 Table — (PDF) [file pone.0202095.s001.pdf]

Supporting Information

S1 Table. Individual characteristics of included (N=3,751) and excluded (N=577) participants

	Included (N=3751)		Excluded (N=576)		*P*[a]
	N	*%*	*N*	*%*	
**Sex**					
**Men**	698	18.61	148	25.69	<0.0001
**Women**	3053	81.39	428	74.31	
**Age (year)**					
**18-29**	265	7.06	22	3.82	<0.0001
**30-49**	1595	42.52	166	28.82	
**50-64**	1128	30.07	196	34.03	
**≥65**	763	20.34	192	33.33	
**Educational level**					
**Up to secondary**	505	13.46	116	20.14	<0.0001
**Some college**	477	12.72	76	13.19	
**University degree**	2730	72.78	370	64.24	
**Missing data**	39	1.04	14	2.43	
**Monthly income per consumption unit (€/CU)**					
**<1200**	20	6.40	27	4.69	0.5
**1200-1800**	554	14.77	91	15.80	
**1800-2700**	497	13.25	84	14.58	
**>2700**	2041	54.41	308	53.47	
**Missing data**	419	11.17	66	11.46	
**Household composition**					
**Adults only**	2521	67.21	409	71.01	0.07
**Adults and children**	1230	32.79	167	28.99	
**Smoking status**					
**Current smoker**	561	14.96	87	15.10	0.9
**Former smoker**	1246	33.22	195	33.85	
**Never smoker**	1944	51.83	294	51.04	
**Physical activity level**					
**High**	1137	30.31	216	37.50	0.004
**Moderate**	1658	44.20	225	39.06	
**Low**	929	24.77	129	22.40	
**Missing data**	27	0.72	6	1.04	
**Body Mass Index (kg/m²)**					
**<18.5**	209	5.57	29	5.03	0.8
**18.5-24.9**	2370	63.18	367	63.72	
**25-29.9**	810	21.59	132	22.92	
**30-34.9**	266	7.09	33	5.73	
**35-39.9**	68	1.81	11	1.91	
**≥40**	28	0.75	4	0.69	

		**mPNNS-GS**			
**Quartile 1**	811	21.62	114	19.79	0.3
**Quartile 2**	812	21.65	15	19.97	
**Quartile 3**	819	21.83	125	21.70	
**Quartile 4**	792	21.11	144	25.00	
**Missing data**	517	13.78	78	13.54	

[a] P-values are based on chi-square test.

mPNNS-GS, modified “Programme National Nutrition Santé” – Guideline Score; CU, Consumption Unit. One CU is attributed for the first adult of the household, 0.5 CU for other persons aged 14 or older and 0.3 CU for children under 14 years old.
